# Collagen VI Null Mice as a Model for Early Onset Muscle Decline in Aging

**DOI:** 10.3389/fnmol.2017.00337

**Published:** 2017-10-24

**Authors:** Daniele Capitanio, Manuela Moriggi, Sara De Palma, Dario Bizzotto, Sibilla Molon, Enrica Torretta, Chiara Fania, Paolo Bonaldo, Cecilia Gelfi, Paola Braghetta

**Affiliations:** ^1^Department of Biomedical Sciences for Health, University of Milano, Milan, Italy; ^2^CNR-IBFM, Milan, Italy; ^3^Department of Molecular Medicine, University of Padova, Padova, Italy; ^4^UO Proteomica Clinica, IRCCS Policlinico S. Donato, Milan, Italy

**Keywords:** collagen VI, aging muscle proteome, skeletal muscle, autophagy, lipotoxicity

## Abstract

Collagen VI is an extracellular matrix (ECM) protein playing a key role in skeletal muscles and whose deficiency leads to connective tissue diseases in humans and in animal models. However, most studies have been focused on skeletal muscle features. We performed an extensive proteomic profiling in two skeletal muscles (diaphragm and gastrocnemius) of wild-type and collagen VI null (*Col6a1*^−/−^) mice at different ages, from 6- (adult) to 12- (aged) month-old to 24 (old) month-old. While in wild-type animals the number of proteins and the level of modification occurring during aging were comparable in the two analyzed muscles, *Col6a1*^−/−^ mice displayed a number of muscle-type specific variations. In particular, gastrocnemius displayed a limited number of dysregulated proteins in adult mice, while in aged muscles the modifications were more pronounced in terms of number and level. In diaphragm, the differences displayed by 6-month-old *Col6a1*^−/−^ mice were more pronounced compared to wild-type mice and persisted at 12 months of age. In adult *Col6a1*^−/−^ mice, the major variations were found in the enzymes belonging to the glycolytic pathway and the tricarboxylic acid (TCA) cycle, as well as in autophagy-related proteins. When compared to wild-type animals *Col6a1*^−/−^ mice displayed a general metabolic rewiring which was particularly prominent the diaphragm at 6 months of age. Comparison of the proteomic features and the molecular analysis of metabolic and autophagic pathways in adult and aged *Col6a1*^−/−^ diaphragm indicated that the effects of aging, culminating in lipotoxicity and autophagic impairment, were already present at 6 months of age. Conversely, the effects of aging in *Col6a1*^−/−^ gastrocnemius were similar but delayed becoming apparent at 12 months of age. A similar metabolic rewiring and autophagic impairment was found in the diaphragm of 24-month-old wild-type mice, confirming that fatty acid synthase (FASN) increment and decreased microtubule-associated proteins 1A/1B light chain 3B (LC3B) lipidation are hallmarks of the aging process. Altogether these data indicate that the diaphragm of *Col6a1*^−/−^ animal model can be considered as a model of early skeletal muscle aging.

## Introduction

In elderly, muscle weakness and reduced muscle mass are associated with an increased risk of falls, which is in turn a primary cause of bone fracture. However, the mechanisms underlying the muscle weakness and reduced walking speed associated with loss of lean mass, defined as sarcopenia, are still poorly understood. It has been demonstrated that a proper activity of the autophagic process is essential for muscle homeostasis and health and prevents the onset of the frailty syndrome (Xue, 2011; Biolo et al., 2014). Previous studies demonstrated that insufficient level of autophagy and dysregulation of specific metabolic pathways are linked to the onset of a dystrophic phenotype in collagen VI-related myopathies. The same is true for sarcopenia and muscle aging, as autophagy activity is known to decline with aging and a metabolic rearrangement is a characteristic feature of aged muscles.

Animal models are very valuable tools to decipher at the molecular level changes occurring during muscle aging, sarcopenia and muscle disorders. In order to carry out comparative studies between animal models and humans, a precise phenotypic characterization should be performed, accounting for both muscle development and metabolism (Capitanio et al., 2005, 2009, 2016a,b). In mice muscle fiber type distribution and contractile properties are different, as well as metabolism, which in mice is five-time faster compared to humans. Therefore, it becomes clear that in order to translate results from human muscle into an animal model several parameters should be carefully considered, including age, muscle type, training/detraining and feeding.

In previous studies, we demonstrated that the diaphragm of collagen VI null (*Col6a1*^−/−^) mice is the best candidate to analyze the cellular and molecular disease defects of collagen VI-related myopathies, since among all muscles diaphragm is the tissue that better recapitulates the human phenotype (Bonaldo et al., 1998; Irwin et al., 2003; Grumati et al., 2010).

Collagen VI is a remarkable member of the collagen family, due to its broad and dynamically regulated expression and its characteristic network of beaded microfilaments in the extracellular matrix (ECM; Cescon et al., 2015). In muscle, the microfibrillar network of collagen VI surrounds the basement membrane of myofibers, binding other matrix components and transferring mechanical and biochemical signals from the extracellular environment to muscle cells. Collagen VI is also present in the interstitial space of many other tissues including tendon, skin, cartilage, intestine, nervous system and intervertebral discs (Cescon et al., 2015). Mutations of the genes coding for collagen VI polypeptide chains are causative of a broad spectrum of diseases in humans, including Bethlem myopathy and Ullrich congenital muscular dystrophy. Disorders caused by mutations of collagen VI genes mainly affect muscle and connective tissues, leading to muscle weakness, joint laxity, contractures and abnormal skin (Lampe and Bushby, 2005; Bönnemann, 2011). Initial studies aimed at dissecting the molecular mechanisms underlying muscle defects in collagen VI deficient mice revealed an increased apoptosis, associated with organelle alterations and mitochondrial dysfunction, due to an abnormal increased opening of the permeability transition pore (PTP) within the mitochondrial inner membrane (Irwin et al., 2003; Bernardi and Bonaldo, 2008). Later, further studies allowed ascertaining that these defects are linked to an impairment of autophagy, which in turn exacerbates the myofiber dysfunction by preventing the clearance of defective organelles (Grumati et al., 2010, 2011). Together, these defects lead to myofiber apoptosis and muscle wasting, which are characteristic also of patients affected by collagen VI myopathies (Irwin et al., 2003; Grumati et al., 2011). Further studies suggested that alterations of the sarcolemma (the barrier and the link between ECM and intracellular environment of muscle fibers) might be also involved in the myofiber dysfunction triggered by collagen VI deficiency (Canato et al., 2010). By using an untargeted proteomic approach, metabolic and protein phenotypic alterations could be identified in gastrocnemius, tibialis anterior and particularly in diaphragm muscles of *Col6a1*^−/−^ mice (De Palma et al., 2013). *Col6a1*^−/−^ diaphragm is characterized by decreased glycolysis and changes of the tricarboxylic acid (TCA) cycle fluxes, leading to lipotoxicity. These metabolic changes are also associated with alterations of proteins involved in mechanotransduction at the myotendinous junction/costameric/sarcomeric level (i.e., tenascin C, focal adhesion kinase, rho-associated protein kinase 1, troponin I fast), suggesting a relationship between ECM architectural dysregulation and muscle metabolism in *Col6a1*^−/−^ mice, which was also confirmed in Bethlem myopathy patients (De Palma et al., 2014).

In the present study, we investigated the proteomic changes occurring in the diaphragm and gastrocnemius muscles of *Col6a1*^−/−^ mice during aging, and found that alterations in gluconeogenesis, glycolysis and TCA cycle rewiring led to lipotoxicity and autophagic decline. These alterations were present in the diaphragm of *Col6a1*^−/−^ mice already at 6 months of age, and appeared in gastrocnemius at 12 months of age. Since the same alterations became evident in wild-type animals only at 24 months of age, the data indicate that skeletal muscles of *Col6a1*^−/−^ mice are characterized by a precocious aging, suggesting that they could be used as novel model to investigate muscle aging. Furthermore, in the present study, we identified fatty acid synthase (FASN) and microtubule-associated proteins 1A/1B light chain 3 BII (LC3BII)/LC3BI ratio as new markers of the aging process in muscle tissue.

## Materials and Methods

### Ethics Statement

All the procedures involving animals and their care were carried out in agreement with the institutional procedures in compliance with national regulations (D.L. No. 116, G.U. Suppl. 40, Feb. 18, 1992, Circolare No. 8, G.U., 14, 1994) and international regulations (EEC Council Directive 86/609, OJ L 358, 1 DEC.12, 1987; NIH Guide for the Care and use of Laboratory Animals, U.S. National Research Council, 1996). Mouse procedures were approved by the Ethics Committee of the University of Padova and authorized by the Italian Ministry of Health (Permit Number: 22675). The animals were anesthetized before sacrifice and all efforts were made to minimize suffering.

### Mice and Sample Collection

All the proteomic analyses were performed in 6- and 12-month-old *Col6a1*^−/−^ (collagen VI null) mice and wild-type control mice in the same inbred C57BL/6NCrl strain. Immunoblotting analyses were also performed at 24 months of age. Four male mice per group were used. Mice were housed in individual cages in an environmentally controlled room (23°C, 12-h light-dark cycle) and provided with food and water *ad libitum*. Animals were sacrificed and diaphragm and gastrocnemius muscles were removed and immediately frozen in liquid nitrogen, ground in a dry ice-cooled mortar and stored at −80°C.

### Myosin Heavy Chain (MyHC) Isoform Composition

SDS electrophoresis of the muscle extracts was performed as described (Danieli Betto et al., 1986) in a discontinuous buffer system with a 4%T stacking gel, pH 6.8 and a 6%T, constant concentration, 37% w/v glycerol, pH 8.8, running gel, respectively. Samples (4 μg) were separated at 100 V, overnight. Gels were stained with SYPRO Orange (Molecular probes) and scanned at 570 nm with a Typhoon laser densitometer (GE Healthcare). Quantitation was achieved using ImageQuant (Molecular Dynamics) software. MyHC-specific isoforms were identified by electrospray ionization/tandem mass spectrometry (ESI-MS/MS), as described previously (Moriggi et al., 2010).

### Proteomic Analysis

#### Two-Dimensional Difference in Gel Electrophoresis (2D-DIGE)

Protein labeling, 2D-separation and analysis were performed as previously described (Viganò et al., 2011). The adopted 3–10 non-linear pH gradient enables separation of protein isoforms in the first dimension, providing a detailed pattern of the muscle proteome. The proteomic profiles of diaphragm and gastrocnemius muscles of wild-type and *Col6a1*^−/−^ mice were compared at different ages (12-month-old vs. 6-month-old mice).

#### Protein Identification by MALDI-TOF and ESI Mass Spectrometry

For protein identification, semi preparative gels were loaded with unlabeled sample (400 μg per strip) and the same electrophoretic conditions of two-dimensional difference in gel electrophoresis (2D-DIGE) were applied. Gels were then stained with a total-protein fluorescent stain (Deep purple, GE Healthcare) and images were acquired using a Typhoon 9200 laser scanner. Spots of interest were excised from gel using the Ettan spot picker robotic system (GE Healthcare), destained in 50% methanol/50 mM ammonium bicarbonate (AMBIC) and incubated with 30 μl of 6 ng/μl trypsin (Promega) dissolved in 10 mM AMBIC for 16 h at 37°C. Released peptides were subjected to reverse phase chromatography (Zip-Tip C18 micro, Millipore), eluted with 50% acetonitrile/0.1% trifluoroacetic acid. Peptide mixture (1 μl) was diluted in an equal volume of 10 mg/ml α-cyano-4-hydroxycinnamic acid matrix dissolved in 70% acetonitrile/30% citric acid and processed on a Ultraflex III MALDI-ToF/ToF (Bruker Daltonics) mass spectrometer. Mass spectrometry was performed at an accelerating voltage of 20 kV and spectra were externally calibrated using Peptide Mix calibration mixture (Bruker Daltonics); 1000 laser shots were taken per spectrum. Spectra were processed by FlexAnalysis software v. 3.0 (Bruker Daltonics) setting the signal to noise threshold value to 6 and search was carried out by correlation of uninterpreted spectra to Rodentia entries (133347 sequences) in NCBInr 20100918 (11833178 sequences; 4040378175 residues). The significance threshold was set at a *p*-value < 0.05. No mass and pI constraints were applied and trypsin was set as enzyme. One missed cleavage per peptide was allowed and carbamidomethylation was set as fixed modification while methionine oxidation as variable modification. Mass tolerance was set at 30 ppm for MS spectra. To confirm protein identification, a MS/MS spectrum was collected by Ultraflex III MALDI-ToF/ToF (Bruker Daltonics) mass spectrometer, as acceptance criterium. Spectra were searched against the database using BioTools v. 3.2 (Bruker Daltonics) interfaced to the on-line MASCOT software, which utilizes a robust probabilistic scoring algorithm. The significance threshold was set at a *p-value* < 0.05. One missed cleavage per peptide was allowed and carbamidomethylation was set as fixed modification while methionine oxidation as variable modification. Mass tolerance was set at 30 ppm and 0.5 Da for peptide and MS/MS fragment ion, respectively.

Additional analyses were performed by ESI-MS/MS, in those cases where the above approach revealed to be unsuccessful. MS/MS spectra were recorded using a HCT Ultra mass spectrometer (Bruker Daltonics) interfaced to an EASY-nLC chromatograph (Proxeon). The samples were dissolved in 0.1% aqueous formic acid, injected onto a 0.075 × 100 mm EASY-Column (Proxeon), and eluted with an acetonitrile/0.1% formic acid gradient (from 5% to 50% ACN). The capillary voltage was set to 1600 V, and data-dependent MS/MS acquisitions were performed on precursors with charge states of 2, 3 or 4 over a survey mass range of 300–1500. The collision gas was helium. Proteins were identified by correlation of uninterpreted MS/MS to Rodentia entries in NCBInr, using MASCOT software. No mass and pI constraints were applied and Trypsin was set as enzyme. One missed cleavage per peptide was allowed, and the precursor and fragment ion tolerance window was set to 0.3 Da. Carbamidomethylation of cysteine was set as fixed modification, whereas methionine oxidation as variable modification.

### Immunoblotting

Protein extracts (50 μg) from diaphragm muscles of a pool of 6-, 12- and 24-month-old wild-type and *Col6a1*^−/−^ mice were loaded in duplicate and resolved on 6%, 10% and 12% polyacrylamide gels, according to protein molecular weight. Blots were then incubated with the following antibodies: anti-fructose- 1,6-bisphosphatase (anti-FBP1; Novus biologicals NBP1-95662, 1:500), anti-Hexokinase (anti-HK; Cell Signaling Technology #2024, 1:500), anti-GLUL (Sigma Aldrich, G2781, 1:500), anti-ODC (Santa Cruz Biotechnology sc-34181, 1:1000), anti-GSS (Santa Cruz Biotechnology sc-28966, 1:500), anti-IDH1 (Santa Cruz Biotechnology sc-49996, 1:500), anti-FASN (Cell Signaling Technology #3189, 1:500), anti-Beclin 1 (Cell Signaling Technology #3738, 1:1000) and anti-LC3B (Cell Signaling Technology #2775 1:1000). After washing, membranes were incubated with anti-rabbit (GE Healthcare), anti-mouse or anti-goat (Santa Cruz Biotechnology) secondary antibodies conjugated with horseradish peroxidase. Signals were visualized by chemiluminescence using the ECL Plus detection kit and the ImageQuant LAS 4000 mini (GE Healthcare) digital imaging system.

### Statistical Analysis

#### 2D-DIGE

Statistically significant differences of 2D-DIGE data were computed by an unpaired Student’s *t*-test (*p* < 0.01). False discovery rate (FDR) was applied as a multiple test correction in order to keep the overall error rate as low as possible. Power analysis was conducted on statistically changed spots, and only spots that reached a sensitivity threshold >0.8 were considered as differentially expressed.

#### MyHC Quantitation

Bands were detected and quantitated using an image analysis software (ImageQuant, Molecular Dynamics). The intensity of each MyHC band in a lane was normalized against the sum of the band intensities for that lane and then multiplied by 100 to obtain the % composition. Statistical analysis was performed by applying unpaired Student’s *t*-test (between *Col6a1*^−/−^ and wt) or independent one-way analysis of variance (ANOVA) and Tukey (among the 3 timepoints), *n* = 2, *p* < 0.05.

#### Immunoblotting

Image analysis (ImageQuant TL, Molecular Dynamics) was performed followed by statistical analysis by applying unpaired Student’s *t*-test (between *Col6a1*^−/−^ and wt) or independent one-way ANOVA and Tukey (among the 3 timepoints), *n* = 2, *p* < 0.05. Band intensities were normalized against the total amount of proteins stained by Sypro Ruby.

### Histological Staining

Gastrocnemius and diaphragm muscles from *Col6a1*^−/−^ and control mice of 6, 12 and 24 months of age were dissected, frozen in liquid nitrogen and embedded in OCT medium prior to cryosectioning. Ten micrometer thick sections were fixed in PFA 4% at RT for 10 min and stained with ORO working solution (Oil Red O stock solution (Sigma #O1391) diluted in water to 0.36% final) for 10 min at RT. The sections were rinsed in running tap water for 30 min and then counterstained with Modified Weigert’s Iron Hematoxylin (Sigma cat. # HT1079), following manufacturer’s protocol.

## Results

### Proteomic Profile of Diaphragm and Gastrocnemius Muscles during Aging in Wild-Type and *Col6a1*^−/−^ Mice

The proteomic profiles of diaphragm and gastrocnemius muscles of 12-month-old wild-type and *Col6a1*^−/−^ mice were compared to the respective 6-month-old animals to highlight changes occurring in aging. In wild-type samples, 36 spots in diaphragm and 40 spots in gastrocnemius were changed during aging, and among them 20 spots showed a common trend between the two muscles. In *Col6a1*^−/−^ mice, 30 spots were changed in diaphragm and 46 in gastrocnemius, and among them 8 spots were found to be commonly dysregulated in the two muscles. All the proteins that we identified to be changed are cited in the text and figures using their gene name and are listed in supporting information together with their UniProtKB accession number and MALDI-ToF/ToF MS data (Supplementary Table S1). We subgrouped proteins into the following classes: structural/contractile, metabolic and stress response proteins.

#### Structural/Contractile Proteins

**Figure 1:**
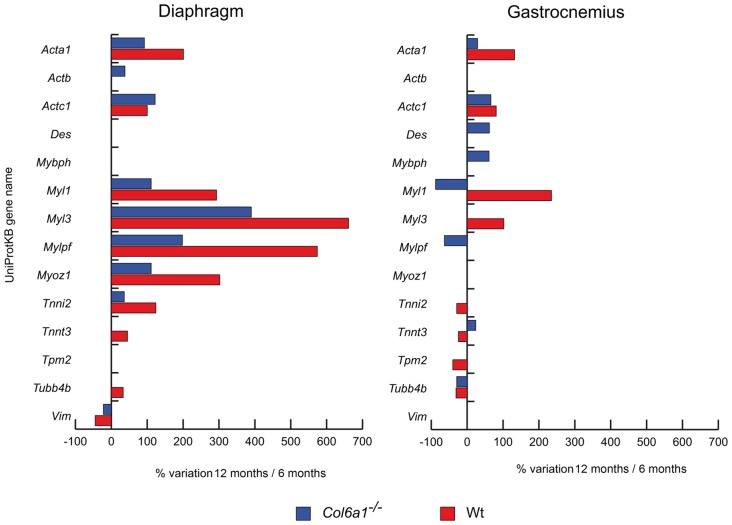
Histograms of differentially expressed contractile/structural proteins in *Col6a1*^−/−^ (blue bars) and wild-type (Wt) mice (red bars). Isoforms of proteins significantly altered (Student’s *t*-test, *n* = 4, *p* < 0.01) are expressed as % of spot volume variation in 12-month-old vs. 6-month-old mice (*Acta1*: skeletal muscle alpha actin; *Actb*: cytoplasmic actin; *Actc1*: cardiac alpha actin; *Des*: desmin; *Mybph*: myosin binding protein h; *Myl1*: myosin light chain 1/3; *Myl3*: myosin light chain 3; *Mylpf*: myosin regulatory light chain 2; *Myoz1*: myozenin-1; *Tnni2*: troponin I; *Tnnt3*: troponin T fast; *Tpm2*: tropomyosin beta chain; *Tubb4b*: tubulin-beta-2C chain; *Vim*: vimentin).

Structural and contractile protein variations at 12 months, compared to 6 months of age, are shown in Figure 1. In both wild-type and *Col6a1*^−/−^ diaphragms skeletal alpha actin (*Acta1*), cardiac alpha actin (*Actc1*), myosin light chain 1/3 (*Myl1*), myosin light chain 3 (*Myl3*), myosin regulatory light chain 2 (*Mylpf*), myozenin-1 (*Myoz1*) and troponin I (*Tnni2*) showed an increment of their protein levels, whereas vimentin (*Vim*) showed a decrease. *Col6a1*^−/−^ aged diaphragms showed an increment of cytoplasmic actin (*Actb*), whereas wild-type diaphragms showed an increment of troponin T fast (*Tnnt3*) and tubulin-beta-2C chain (*Tubb4b*). In gastrocnemius, both wild-type and *Col6a1*^−/−^ animals displayed increased levels of *Acta1* and *Actc1*, and a decrease in *Tubb4b*. *Myl1* decreased in *Col6a1*^−/−^ and increased in wild-type mice, whereas *Tnnt3* was increased in *Col6a1*^−/−^ and decreased in wild-type. Desmin (*Des*) and myosin binding protein h (*Mybph*) increased only in *Col6a1*^−/−^ gastrocnemius, whereas *Mylpf* decreased. *Myl3* increased in wild-type, whereas *Tnni2* and tropomyosin beta chain (*Tpm2*) decreased.

#### Metabolic Proteins

Dysregulated metabolic proteins were sub-grouped into different classes: glycolysis and glucose metabolism, oxidative metabolism and energy homeostasis.

##### Glycolysis and glucose metabolism (Figure 2)

In aged diaphragm, the glycolytic and glycogen biosynthetic proteins fructose-bisphosphate aldolase A (*Aldoa*), beta-enolase (*Eno3*) and triosephosphate isomerase (*Tpi1*) were increased in both wild-type and *Col6a1*^−/−^ mice, whereas phosphoglucomutase 2 (*Pgm2*) decreased. Glyceraldehyde-3-phosphate dehydrogenase (*Gapdh*) and L-lactate dehydrogenase B chain (*Ldhb*) were increased only in wild-type animals. In gastrocnemius, alpha-enolase (*Eno1*), *Eno3*, *Gapdh*, phosphoglycerate mutase (*Pgam2*), *Tpi1* were decreased in *Col6a1*^−/−^ and increased in wild-type mice. A common decrement of *Pgm2* was observed. *Aldoa* and phosphoglycerate kinase 1 (*Pgk1*) decreased and *Ldhb* increased only in *Col6a1*^−/−^ mice.

**Figure 2:**
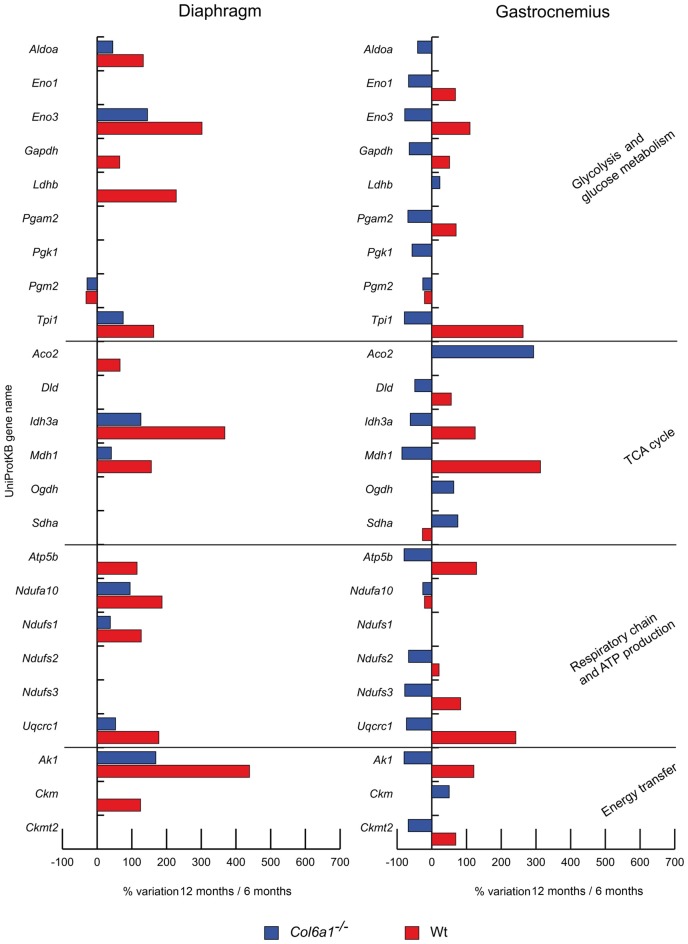
Histograms of differentially expressed metabolic proteins in *Col6a1*^−/−^ (blue bars) and Wt mice (red bars). Isoforms of proteins significantly altered (Student’s *t*-test, *n* = 4, *p* < 0.01) are expressed as % of spot volume variation in 12-month-old vs. 6-month-old mice (*Aldoa*: aldolase A; *Eno1*: enolase; *Eno3*: beta-enolase; *Gapdh*: glyceraldehyde-3-phosphate dehydrogenase; *Ldhb*: L-lactate dehydrogenase B; *Pgam2*: phosphoglycerate mutase; *Pgk1*: phosphoglycerate kinase 1; *Pgm2*: phosphglucomutase 2; *Tpi1*: triosephosphate isomerase 1; *Aco2*: aconitate hydratase; *Dld*: dihydrolipoamide dehydrogenase 1; *Idh3a*: isocitrate dehydrogenase alpha subunit; *Mdh1*: cytoplasmic malate dehydrogenase; *Ogdh*: oxoglutarate dehydrogenase; *Sdha*: succinate dehydrogenase; *Atp5b*: ATP synthase subunit beta; *Etfa*: electron transfer flavoprotein subunit alpha; *Ndufa10*: NADH dehydrogenase [ubiquinone] 1 alpha subcomplex subunit 10; *Ndufs1*: NADH-ubiquinone oxidoreductase 75 kDa subunit; *Ndufs2*: NADH-dehydrogenase iron-sulfur protein 2; *Ndufs3*: NADH-dehydrogenase iron-sulfur protein 3; *Uqcrc1*: ubiquinol-cytochrome c reductase core protein I; *Ak1*: adenylate kinase 1; *Ckm*: creatine kinase; *Ckmt2*: mitochondrial creatine kinase S-type).

##### Oxidative metabolism (Figure 2)

The TCA cycle proteins isocitrate dehydrogenase alpha subunit (*Idh3a*) and cytoplasmic malate dehydrogenase (*Mdh1*) increased in both wild-type and *Col6a1*^−/−^ diaphragms, together with the respiratory chain complex I NADH dehydrogenase [ubiquinone] 1 alpha subcomplex subunit 10 (*Ndufa10*) and NADH-ubiquinone oxidoreductase 75 kDa subunit (*Ndufs1*) and the complex III ubiquinol-cytochrome c reductase core protein I (*Uqcrc1*). Aconitate hydratase (*Aco2*) and ATP synthase subunit beta (*Atp5b*) increased only in wild-type animals. In aged gastrocnemius, *Ndufa10* decreased in both wild-type and *Col6a1*^−/−^ mice, whereas dihydrolipoamide dehydrogenase 1 (*Dld*), *Idh3a*, *Mdh1*, *Atp5b*, NADH dehydrogenase iron-sulfur protein 2 and 3 (*Ndufs2*, *Ndufs3*), and *Uqcrc1* decreased in *Col6a1*^−/−^ and increased in wild-type; at variance, succinate dehydrogenase (*Sdha*) showed an opposite trend. *Aco2* and oxoglutarate dehydrogenase (*Ogdh*) were increased in *Col6a1*^−/−^ mice.

##### Energy homeostasis (Figure 2)

In aged *Col6a1*^−/−^ diaphragm, only adenylate kinase-1 (*Ak1*) resulted increased, whereas *Ak1* and creatine kinase (*Ckm*) increased in wild-type. In aged gastrocnemius, *Ak1* and mitochondrial creatine kinase S-type (*Ckmt2*) decreased in *Col6a1*^−/−^ and increased in wild-type. *Ckm* increased in *Col6a1*^−/−^ only.

#### Stress Response, Folding and Endoplasmic Reticulum Trafficking Proteins

**Figure 3:**
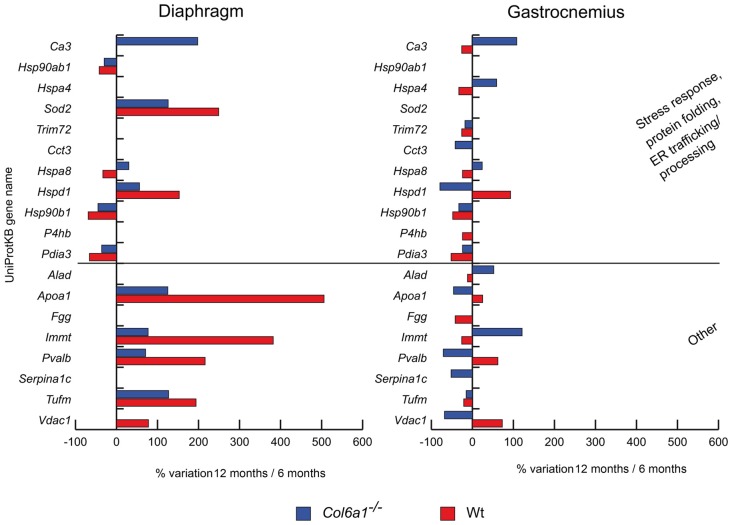
Histograms of differentially expressed stress response and other proteins in *Col6a1*^−/−^ (blue bars) and Wt mice (red bars). Isoforms of proteins significantly altered (Student’s *t*-test, *n* = 4, *p* < 0.01) are expressed as % of spot volume variation in 12-month-old vs. 6-month-old mice (*Ca3*: carbonic anhydrase 3; *Hsp90ab1*: heat shock protein HSP 90-beta; *Hspa4*: heat shock 70 kDa protein 4; *Sod2*: mitochondrial superoxide dismutase [Mn]; *Trim72*: tripartite motif-containing protein 72; *Cct3*: T-complex protein 1 subunit gamma; *Hspa8*: heat shock cognate 71 kDa protein; *Hspd1*: heat shock protein 60 kDa; *Hsp90ab1*: heat shock protein HSP 90-beta; *P4hb*: protein disulfide-isomerase; *Pdia3*: protein disulfide isomerase A3; *Alad*: delta-aminolevulinic acid dehydratase; *Apoa1*: apolipoprotein A-I; *Fgg*: fibrinogen gamma; *Immt*: mitochondrial contact site complex subunit MIC60; *Pvalb*: parvalbumin; *Serpina1c*: alpha-1-antitrypsin 1-3; *Tufm*: mitochondrial elongation factor Tu; *Vdac1*: voltage-dependent anion-selective channel protein 1).

In aged diaphragm, heat shock protein HSP 90-beta (*Hsp90ab1*), endoplasmin (*Hsp90b1*) and protein disulfide-isomerase A3 (*Pdia3*) decreased in both wild-type and *Col6a1*^−/−^ mice (Figure 3). Conversely, mitochondrial superoxide dismutase (*Sod2*) and heat shock protein 60 kDa (*Hspd1*) increased. Heat shock cognate 71 kDa protein (*Hspa8*) increased in *Col6a1*^−/−^ and decreased in wild-type, whereas carbonic anhydrase 3 (*Ca3*) increased only in *Col6a1*^−/−^. In gastrocnemius of both wild-type and *Col6a1*^−/−^ *Hsp90b1*, *Pdia3* and tripartite motif-containing protein 72 (*Trim72*) were decreased. *Ca3*, heat shock 70 kDa protein 4 (*Hspa4*) and *Hspa8* display a different regulation, being increased in *Col6a1*^−/−^ and decreased in wild-type gastrocnemius. On the contrary, *Hspd1* decreased in *Col6a1*^−/−^ and increased in wild-type gastrocnemius. Protein disulfide-isomerase (*P4hb*) decreased in wild-type, whereas T-complex protein 1 subunit gamma (*Cct3*) decreased in *Col6a1*^−/−^ only.

#### Other Proteins

We also identified other proteins with different roles, dysregulated during aging in *Col6a1*^−/−^ mice. Notably, the majority of them were mitochondrial proteins (Figure 3). In aged diaphragms, apolipoprotein A-I (*Apoa1*), the mitochondrial contact site complex subunit MIC60 (*Immt*), parvalbumin (*Pvalb*), and the mitochondrial elongation factor Tu (*Tufm*) increased in both wild-type and *Col6a1*^−/−^ mice. The voltage-dependent anion-selective channel protein 1 (*Vdac1*) increased in wild-type only. In gastrocnemius, only *Tufm* decreased in both wild-type and *Col6a1*^−/−^ mice. *Apoa1*, *Pvalb* and *Vdac1* decreased in *Col6a1*^−/−^ and increased in wild-type, whereas delta-aminolevulinic acid dehydratase (*Alad*) and *Immt* were counter-regulated. Fibrinogen gamma (*Fgg*) decreased in wild-type, whereas alpha-1-antitrypsin 1-3 (*Serpina1c*) decreased in *Col6a1*^−/−^ only.

### Diaphragm Characterization

Since aging traits appear prominent in the *Col6a1*^−/−^ diaphragm at 6 months of age, knowing that changes in myosin heavy chain (MyHC) composition, in molecules regulating the carbon source utilization and autophagy are dysregulated in *Col6a1*^−/−^ adult mice compared to adult wild-type (De Palma et al., 2013), these molecules were assessed by immunoblotting in this muscle.

#### Myosin Heavy Chain Isoform Composition

**Figure 4:**
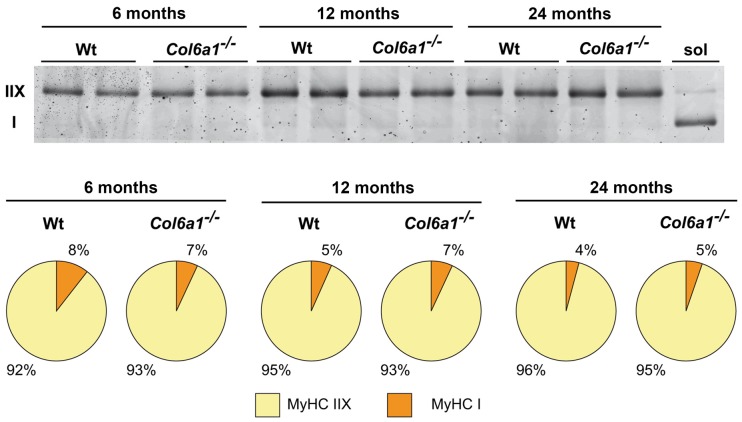
Myosin heavy chain (MyHC) fiber type composition (%) of Wt and *Col6a1*^−/−^ diaphragms. Samples (4 μg) were run in duplicate at 100 V overnight. Soleus muscle (sol) was loaded as control for MyHC I band detection. Gel was SYPRO Orange (Molecular Probes) stained, and images were acquired at 570 nm on a Typhoon 9200 laser scanner. Protein band quantification was achieved using ImageQuant software (Molecular Dynamics).

In diaphragm, the distribution of MyHC isoforms I and IIX was comparable between wild-type and *Col6a1*^−/−^ mice of the same age (Figure 4). Diaphragm was characterized by a prevalence of MyHC-IIX/A fast-twitch oxidative fibers (92%–96%), followed by slow-twitch oxidative MyHC-I fibers (4%–8%). The regenerating MyHC-IIB (fast-twitch glycolytic) fibers were undetectable. Moreover, our results show a progressive decline, although not supported by statistics, of slow fibers in wild-type during aging (50% decrement from 6 to 24 months), that is less prominent in the absence of collagen VI (35% decrement).

#### Alpha-Ketoglutarate Rewiring in Aging

**Figure 5:**
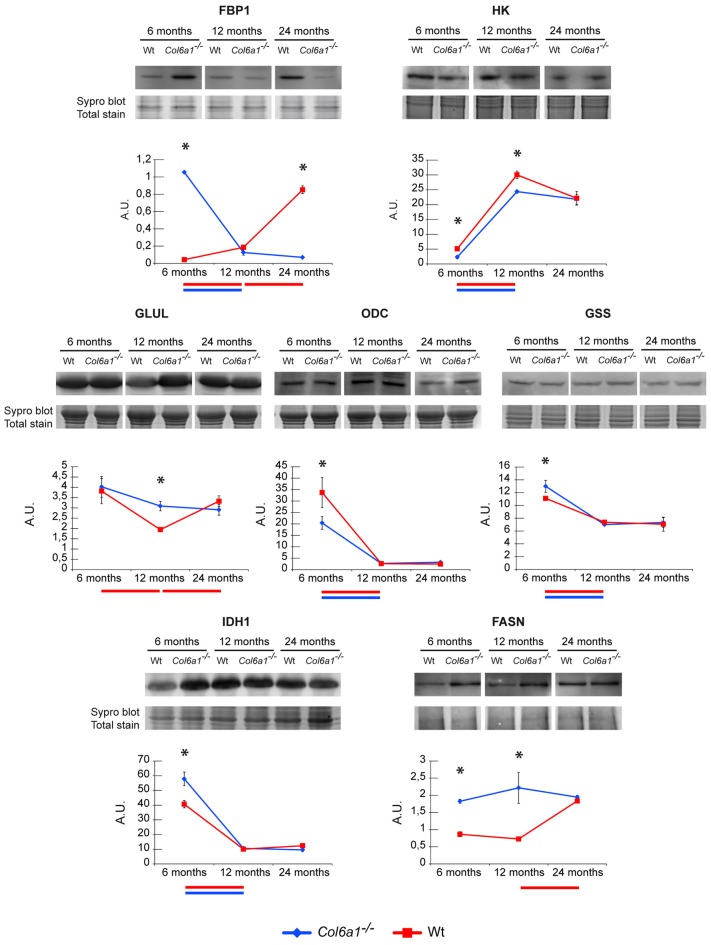
Representative line charts and immunoblotting images of fructose-1,6-bisphosphatase (FBP1), Hexokinase (HK), GLUL, ornithine decarboxylase (ODC), glutathione synthetase (GSS) and IDH1, fatty acid synthase (FASN), in *Col6a1*^−/−^ (blue line) and Wt (red line) diaphragm at 6, 12 and 24 months of age. Blot images have been spliced for a better visualization, full-length images are available in supplementary material (Supplementary Figures S1A–C,E,G–I). Variations are expressed as band intensities (arbitrary units). The asterisks mark significant differences in *Col6a1*^−/−^ mice compared to the respective age-matched Wt mice (Student’s *t*-test; *n* = 2; *p < 0.05). Bars below the charts represent significant differences (ANOVA and Tukey; *n* = 2; *p* < 0.05) between timepoints in Wt (red) and *Col6a1*^−/−^ (blue) mice.

In the absence of glucose, glutamine is the major substrate available to cells (Metallo et al., 2012). In skeletal muscle, glutamine can act as a nitrogen donor in the synthesis of proteins and nucleosides (Hammarqvist et al., 1989). It can also be converted to α-ketoglutarate (α-KG), which can anaplerotically support the TCA cycle or be reductively transformed by cytosolic IDH1 to generate citrate (Des Rosiers et al., 1995; Rennie et al., 1996; Yoo et al., 2008). Previous studies demonstrated that in the diaphragm of adult *Col6a1*^−/−^ vs. wild-type mice a decreased glycolytic flux, PDH, IDH3 and α-ketoglutarate dehydrogenase enzyme levels, suggested a severe metabolic imbalance. The decrement of TCA cycle enzymes indicated that the excess of citrate not utilized by the TCA cycle was exported to the cytosol to sustain the synthesis of lipids, as indicated by lipid droplets accumulation among muscle fibers (lipotoxicity; De Palma et al., 2013). Histological analyses on cryosections of diaphragm and gastrocnemius muscle stained with Oil Red O techniques confirmed the presence of lipid droplets in *Col6a1*^−/−^ diaphragms already at 6 months of age, while in control animals the red staining was quite scarce at 12 months of age and became evident at 24 months of age (Supplementary Figure S2A). In gastrocnemius the staining was evident starting at 12 months of age in *Col6a1*^−/−^, while it was almost absent in control samples (Supplementary Figure S2B). Proteomic results showed that this rewiring occurs in diaphragm in 6-month-old mice, whereas in gastrocnemius it persists in 12-month-old mice, suggesting it is a signature of *Col6a1*^−/−^ mouse model. To determine whether TCA cycle rewiring is a peculiarity of collagen VI deficiency or aging, the α-KG fate was investigated in 6-, 12- and in 24-month-old *Col6a1*^−/−^ and wild-type mice (Figure 5). HK and FBP1 levels were evaluated. In 6-month-old *Col6a1*^−/−^ diaphragm an increment of FBP1 and a decrement of HK were observed. In 12-month-old *Col6a1*^−/−^ mice HK remained decreased, and FBP1 was unchanged, whereas in 24-month-old mice these proteins displayed an opposite trend, suggesting a different utilization of carbon sources by *Col6a1*^−/−^ and wild-type diaphragm muscles over time.

In particular, our attention was focused on the reductive/lipogenic pathway (isocitrate dehydrogenase 1, IDH1 and FASN), on glutamine biosynthesis (glutamine synthetase, GLUL), on glutathione biosynthesis (glutathione synthetase, GSS) and on the first step of polyamines biosynthetic pathway (ornithine decarboxylase, ODC). The expression of GLUL, GSS, IDH1, FASN and ODC was evaluated by immunoblotting (Figure 5). In 6-month-old *Col6a1*^−/−^ mice an increment of IDH1 and FASN was observed, ODC decreased, whereas GLUL and GSS were unchanged. In 12-month-old mice GLUL increased and FASN remained increased in *Col6a1*^−/−^ compared with wild-type, whereas IDH1, GSS and ODC were at the same level of 12-month-old wild-type mice. Notably, 24-month-old wild-type mice were characterized by levels of ODC, GLUL and HK similar to 12-month-old *Col6a1*^−/−^, whereas FBP1 levels were similar to 6-month-old *Col6a1*^−/−^, and high levels of FASN both in *Col6a1*^−/−^ and wild-type suggesting that glutamine production became relevant with aging, ending up in lipotoxicity at 6 months of age in *Col6a1*^−/−^ diaphragm and at 24 months of age in wild-type diaphragm.

#### Autophagy

**Figure 6:**
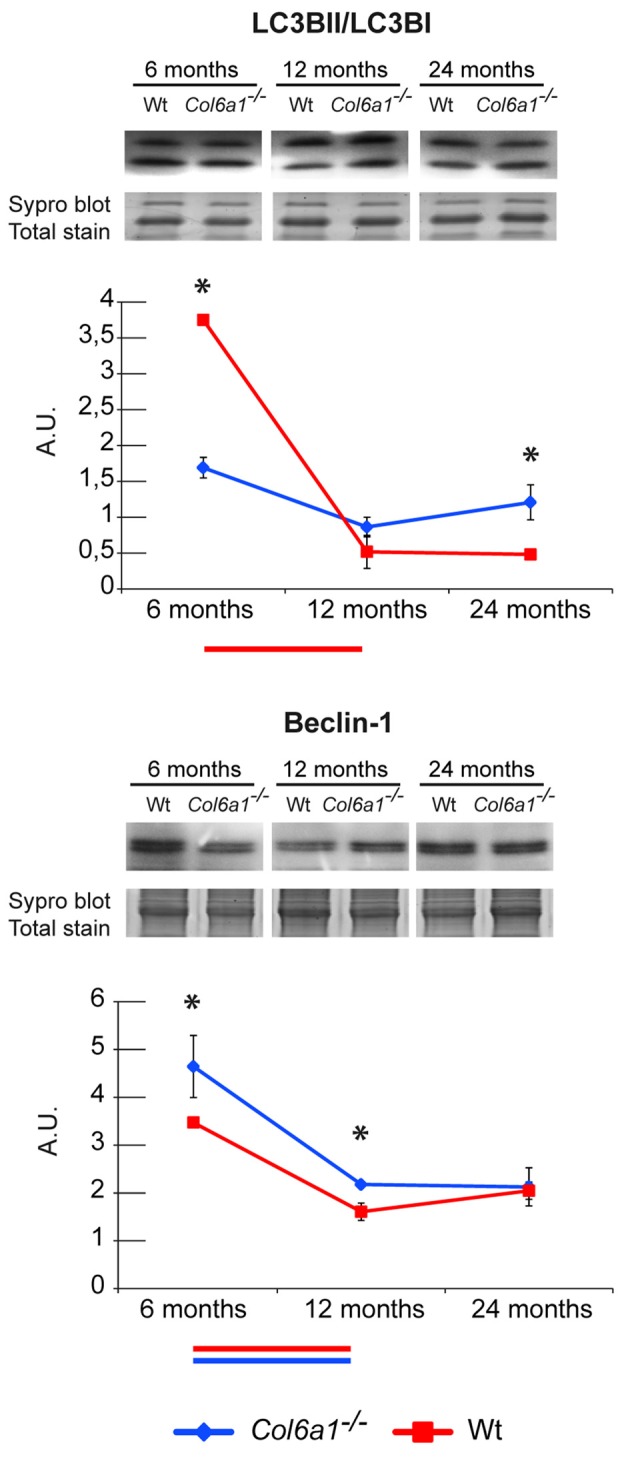
Representative line charts and immunoblotting images of microtubule-associated proteins 1A/1B light chain 3 B (LC3B) and Beclin-1 in 6-, 12- and 24-month-old *Col6a1*^−/−^ (blue line) and wt (red line) diaphragm. Blot images have been spliced for a better visualization, full-length images are available in supplementary material (Supplementary Figures S1D,F). Variations are expressed as band intensities (arbitrary units). The asterisks mark significant differences in *Col6a1*^−/−^ mice compared to the respective age-matched Wt mice (Student’s *t*-test; *n* = 2; *p < 0.05). Bars below the charts represent significant differences (ANOVA and Tukey; *n* = 2; *p* < 0.05) between timepoints in Wt (red) and *Col6a1*^−/−^ (blue) mice.

The autophagic flux in *Col6a1*^−/−^ animals was investigated by immunoblotting, and the expression level of Beclin-1 and microtubule-associated proteins 1A/1B light chain 3B (LC3B) were determined. Concerning the LC3B, the ratio of the lipidated (activated) form (LC3BII) over the delipidated one (LC3BI) was assessed. Our results showed that 6-month-old *Col6a1*^−/−^ diaphragm display an impaired autophagic flux (lower LC3BII/LC3BI ratio despite increased Beclin-1 levels compared to the corresponding wild-type animals), confirming previously published data (Grumati et al., 2010). Comparing *Col6a1*^−/−^ and wild-type mice at 6, 12 and 24 months, it emerged a progressive decrement of LC3BII/LC3BI ratio in wild-type over time, whereas no changes in *Col6a1*^−/−^ mice were detected during aging (Figure 6). Concerning Beclin1, a similar trend of its levels was observed both in *Col6a1*^−/−^ and wild-type.

## Discussion

In the present work we investigated protein alterations occurring in skeletal muscles during aging in the presence or absence of collagen VI, an ECM protein whose mutations are causative of different muscle disorders, including Bethlem myopathy and Ullrich congenital muscular dystrophy. We took advantage from *Col6a1*^−/−^ mice, a well-characterized animal model for collagen VI-related disorders and whose myopathic phenotype is characterized by an early onset and very slow progression (Bonaldo et al., 1998), ultrastructural alterations and dysfunction of organelles (Irwin et al., 2003) and autophagy impairment (Grumati et al., 2010). In this study, we analyzed two different muscles, i.e. diaphragm and gastrocnemius, known to be differently affected in mice (Bonaldo et al., 1998; Grumati et al., 2010) and in muscles of patients affected by collagen VI-related disorders (Bethlem and Wijngaarden, 1976; Nonaka et al., 1981; Haq et al., 1999; Jöbsis et al., 1999; Merlini and Bernardi, 2008; Nadeau et al., 2009; Briñas et al., 2010). Moreover, to study the overall proteomic changes induced over time, the analyses were performed at two different ages (6- and 12-month-old mice; Irwin et al., 2003; Grumati et al., 2010). The combination of proteomic and immunoblotting analyses of specific molecules, enabled us to confirm that the metabolic and structural protein alterations in the diaphragm of *Col6a1*^−/−^ mice, previously detected at 6 months of age, are retained at 12 months. Furthermore, this study allowed us to define the timing of proteomic changes occurring in gastrocnemius, in which the alterations displayed by diaphragm are delayed and appears only at month-12. Overall these results provide a broader picture of changes and the connection with aging in these two muscles. Based on the results of this study, the diaphragm of 12-month-old *Col6a1*^−/−^ mice is metabolically characterized by an increase of the TCA cycle and respiratory chain, thereby resulting in an increased energy production. On the contrary, gastrocnemius of 12-month-old *Col6a1*^−/−^ mice is characterized by a blunted glycolytic flux, TCA cycle and respiratory chain activity. Together with the previous findings, pointing out mitochondrial dysfunction (Irwin et al., 2003), our data suggest that the metabolic changes occurring in absence of collagen VI are caused by an altered signal transduction, instead of changes in fiber type distribution which occurs during aging or unloading processes (Gelfi et al., 2006; Moriggi et al., 2010).

The analysis of data obtained by comparing the protein expression profile between aged and adult wild-type mice revealed a similar pattern of variations in diaphragm and gastrocnemius. This suggests that under physiological conditions aging appears in a common manner in these two different muscle types. Conversely, comparison of the proteomic data obtained in *Col6a1*^−/−^ diaphragm and gastrocnemius showed a, different behavior in the two muscles. In particular, *Col6a1*^−/−^ diaphragm shows all the hallmarks of aging already at 6 months, and these characteristics are maintained over time. These data suggest that a premature aging is occurring in diaphragm in the absence of collagen VI, whereas this process is delayed in gastrocnemius until 12 months of age, a difference which is likely related to the continuous use of the respiratory muscles thereby contributing to exacerbate the aging processes. The metabolic rewiring is a specific feature of *Col6a1*^−/−^ muscle, in which a number of changes at the gluconeogenetic, glycolytic and TCA cycle levels is associated to high levels of FASN. These mechanisms are not observed in the wild-type mouse at 12 months of age, whereas FASN increased at month 24.

By literature, it is well known that autophagy decline is a feature of aging (Zhou et al., 2017). Previous studies demonstrated that muscles of *Col6a1*^−/−^ mice are characterized by autophagy defects, confirmed in the present study. Low levels of the LC3B lipidated form observed at month-6 are maintained during aging. Furthermore, proteomic analysis indicated in *Col6a1*^−/−^ adult and aged mice an increment of Hspa8 involved in the chaperone-mediated autophagy. In the present context Hspa8 over-expression can suggest a partial support of the autophagic process, in spite of LC3BII/LC3BI ratio decrement (Viganò et al., 2011; Levett et al., 2015). Conversely, in wild-type animals, Hspa8 is downregulated at 12 compared to 6 months, FASN is not increased at both ages, and LC3B followed a physiological decline.

In wild-type, as aging progresses (month-24), levels of FASN, LC3B and Beclin1 became similar to *Col6a1*^−/−^ mice. These findings point at *Col6a1*^−/−^ mice as model of early aging in diaphragm, since at 6 months these animals recapitulate aging traits observed in wild-type diaphragm at month 24.

In conclusion, the *Col6a1*^−/−^ mice can be considered a valuable model to study aging, or the autophagy-aging-ECM crosstalk using two muscles (diaphragm and gastrocnemius) in which diaphragm is characterized by a premature decline.

## Author Contributions

CG and DC designed the study; DC performed immunoblotting and analyzed the data; ET and CF performed mass spectrometry; SDP and MM, 2-D DIGE; SM and DB maintained mouse colonies and collected tissue samples; DC, CG, PBonaldo and PBraghetta wrote the manuscript.

## Conflict of Interest Statement

The authors declare that the research was conducted in the absence of any commercial or financial relationships that could be construed as a potential conflict of interest. The handling Editor declared a shared affiliation, though no other collaboration, with several of the authors DC, MM, ET, CG.
