# Supplementary material for: Collagen VI Null Mice as a Model for Early Onset Muscle Decline in Aging
Source: Front Mol Neurosci. 2017 Oct 24;10:337. doi: 10.3389/fnmol.2017.00337 (PMC5660719; doi:10.3389/fnmol.2017.00337)
Supplement: Supplementary file 1 [file Presentation_1.pdf]

Supplementary Material

Collagen VI null mice as a model for early onset muscle decline in aging

Daniele Capitanio, Manuela Moriggi, Sara De Palma, Dario Bizzotto, Sibilla Molon, Enrica Toretta, Chiara Fania, Paolo Bonaldo, Cecilia Gelfi, Paola Braghetta*

*** Correspondence:** Paola Braghetta, Department of Molecular Medicine, University of Padova, Via U. Bassi 58/B, I-35131 Padova, Italy. E-mail: braghett@bio.unipd.it

1 Supplementary Table S1. List of proteins identified in muscles of wild-type and *Col6a1*$^{-/-}$ mice by PMF and MS/MS.

Spots were identified by MALDI/MS. To confirm identification, a MS/MS spectrum per protein was collected by MALDI TOF/TOF as acceptance criterium. The asterisk indicates proteins identified by ESI-Ion trap mass spectrometer. M indicates methionine oxidation.

Gene symbol	Description	UniProt KB entry	Calculated MW	Calculated pI	MASCOT protein score	Coverage %	Matched/searched peaks	MSMS	MS/MS score	m/z	D m/z (ppm)	z	Missed cleavage sites
Aco2	Aconitase 2, mitochondrial	Q505P4	82463.88	7.40	315	43.3	30/47	**634**NAVTQEFGPVPDTAR**648**	111	1601.813	13.20	1	
Acta1	Actin, alpha skeletal muscle	P68133	42051.03	5.23	186	50.0	13/27	**158**GYSFVTTAER**167**	78	1130.5649	15.25	1	
Actb	Beta-actin	A1E281	13539.35	5.93	83	45.5	5/11	**75**IWHHTFYNELR**85**	67	1515.781	21.01	1	
Actc1	Actin, alpha cardiac muscle 1	P68033	41784.64	5.23	92	48.3	7/16	**31**AVFPSIVGRPR**41**	34	1198.730	20.44	1	1
Ak1	Adenylate kinase isoenzyme 1	Q9R0Y5	21539.60	5.67	115	58.1	11/29	**48**YGYTHLSTGDLLR**60**	106	1495.774	13.39	1	
Alad	Delta-aminolevulinic acid dehydratase	P10518	36023.54	6.31	107	26.1	8/17	**200**FASCFYGPFR**209**	33	1251.5649	2.68	1	
Aldoa	Fructose-bisphosphate aldolase A	P05064	39224.74	8.40	202	56.0	18/34	**244**FSNEEIAMATVTALR**258**	91	1652.836	2.94	1	
Apoa1	Apolipoprotein A-I *	Q58EV2	23022.10	6.99	481	37.1	7	**157**LQELQGR**163**	30	422.2635	60.90	2	
								**248**TQVQSVIDK**256**	41	509.2901	15.14	2	
								**36**DFANVYVDAVK**46**	61	620.8291	24.24	2	
								**184**TQLAPHSEQMR**194**	45	657.3192	3.40	2	
								**206**SNPTLNEYHTR**216**	58	666.3053	22.91	2	
								**142**VAPLGAELQESAR**154**	87	670.8702	12.12	2	
								**34**VKDFANVYVDAVK**46**	75	734.4286	44.74	2	1
Atp5b	ATP synthase, H+ transporting mitochondrial F1 complex, beta subunit	Q8CI65	51749.20	4.99	295	51.0	24/29	**226**AHGGYSVFAGVGER**239**	116	1406.668	8.70	1	
Ca3	Carbonic anhydrase 3	P16015	29235.08	6.97	118	53,1	10/36	**68**VVFDDTYDR**76**	69	1129.526	8.83	1	
Cct3	T-complex protein 1 subunit gamma	P80318	60629.98	6.28	112	23.6	11/23	**377**GATQQILDEAER**388**	50	1330.6764	12.52	1	
Ckm	Muscle creatine kinase	P07310	43044.97	6.58	281	48.8	20/24	**87**DLFDPIIQDR**96**	41	1231.642	8.32	1	
Ckmt2	Creatine kinase S-type, mitochondrial	Q6P8J7	47473.32	8.64	125	27.8	12/25	**311**LGYILTCPSNLGTGLR**326**	91	1734.9216	0.51	1	
Des	Desmin	P31001	53366.59	5.21	330	51.6	28/38	**355**FASEANGYQDNIAR**368**	102	1555.735	13.78	1	
Dld	Dihydrolipoyl dehydrogenase, mitochondrial precursor	O08749	54272.35	7.99	155	21.8	11/14	**483**VCHAHPTLSEAFR**495**	74	1524.7380	0.26	1	
Eno1	Enolase 1 (alpha) *	Q5XKE1	47140.82	6.37	705	34.6	12	**413**IEEELGSK**420**	39	452.7466	26.20	2	
								**82**VNVVEQEK**89**	43	472.7881	67.88	2	
								**336**SCNCLLLK**343**	35	504.2518	4.88	2	
								**184**IGAEVYHNLK**193**	45	572.2824	50.81	2	
								**61**GVSQAVEHINK**71**	29	591.3211	6.44	2	
								**93**LMIEMDGTENK**103**	73	640.7659	48.05	2	
								**82**VNVVEQEKIDK**92**	55	650.8480	67.88	2	1
								**16**GNPTVEVDLYTAK**28**	61	703.8650	4.64	2	
								**359**LAQSNGWGVMVSHR**372**	28	519.9584	65.12	3	
								**344**VNQIGSVTESLQACK**358**	90	817.4344	24.54	2	
								**90**IDKLMIEMDGTENK**103**	26	557.2452	561.78	3	
								**203**DATNVGDEGGFAPNILENK**221**	77	980.9988	33.46	2	
Eno3	Enolase 3 (beta, muscle)	Q4FK59	46997.77	6.29	201	40.3	14/17	**359**LAQSNGWGVMVSHR**372**	88	1541.790	16.76	1	

Fgg	Fibrinogen, gamma polypeptide	Q8VCM7	46671.09	5.55	130	40.8	13/34	**188**ESGLYFIRPLK**198**	44	1322.752	4.01	1	1
Gapdh	Glyceraldehyde-3-phosphate dehydrogenase	Q569X2	35678.82	8.45	130	37.5	10/23	**308**LISWYDNEYGYSNR**321**	106	1779.836	21.81	1	
Hsp90ab1	Heat shock protein 84b * HSP90 BETA	P11499	83281.23	4.96	90	4.0	2	**42**ELISNASDALDKIR**55**	36	773.0000	106.64	2	1
								**205**HSQFIGYPITLYLEK**219**	36	905.0000	19.10	2	
Hsp90b1	Endoplasmin *	P08113	90096.75	4.72	566	28.1	21	**44**TDDEVVQR**51**	60	481.3000	139.42	2	
								**88**LIINSLYK**95**	24	482.4000	212.87	2	
								**725**SGYLLPDTK**733**	31	497.3000	67.95	2	
								**349**EVEEDEYK**356**	41	520.8000	144.84	2	
								**76**FAFQAEVNR**84**	46	541.3000	46.37	2	
								**494**LGVIEDHSNR**503**	50	570.4000	186.15	2	
								**548**EAESSPFVER**557**	20	575.8000	47.96	2	
								**385**SILFVPTSAPR**395**	52	594.4000	96.26	2	
								**103**ELISNASDALDK**114**	73	638.4000	117.51	2	
								**547**KEAESSPFVER**557**	47	639.9000	125.23	2	1
								**672**DISTNYYASQK**682**	46	645.4000	148.67	2	
								**435**GVVDSDDLPLNVSR**448**	84	743.4000	25.80	2	
								**512**FQSSHHSTDITSLDQYVER**530**	40	750.7000	19.22	3	
								**253**EEASDYLELDTIK**265**	79	763.4000	43.17	2	
								**143**NLLHVTDTGVGMTR**156**	43	765.4000	12.20	2	
								**103**ELISNASDALDKIR**116**	41	773.0000	106.64	2	1
								**416**VFITDDFHDMMPK**428**	32	814.4000	48.89	2	
								**52**EEEAIQLDGLNASQIR**67**	101	893.5000	53.16	2	
								**304**EESDDEAAVEEEEEEK**319**	96	933.9000	36.42	2	
								**286**TETVEEPLEEDEAAKEEK**303**	62	1038.5000	20.43	2	1
								**304**EESDDEAAVEEEEEEEKKPK**322**	20	1110.5000	11.41	2	1
Hspa4	Heat shock protein 4 HS 70KDA PROTEIN 4	Q3U2G2	94208.73	5.13	146	27.2	16/29	**621**NAVEEYVYEMR**631**	36	1402.6350	3.04	1	
Hspa8	Heat shock protein 8	Q504P4	68778.77	5.37	250	44.9	23/40	**138**TVTNAVVTVPAYFNDSQR**155**	110	1982.0060	4.15	1	
Hspd1	Heat shock protein 1 (chaperonin)	Q8C2C7	57925.78	5.35	104	28.6	11/29	**61**TVIIEQSWGSPK**72**	74	1344.730	3.15	1	
Idh3a	Isocitrate dehydrogenase [NAD] subunit alpha, mit prec	Q9D6R2	36707.29	5.60	156	39.9	16/35	**179**IAEFAFEYAR**188**	70	1216.612	20.80	1	
Immt	Inner membrane protein, mitochondrial	Q8CAQ8	83900.08	6.18	307	45.5	22/26	**391**FVNQLKGESR**400**	44	1177.636	2.21	1	1
Ivd	Isovaleryl-CoA dehydrogenase, mitochondrial	Q9JHI5	42971.36	6.29	147	42.0	14/29	**273**GVYVLMSGLDLER**285**	73	1451.756	0.19	1	
Ldhb	L-lactate dehydrogenase B chain	P16125	36572.30	5.70	183,0	35.6	14/19	**158**VIGSGCNLDSAR**169**	75	1248.6121	9.60	1	
Mdh1	Malate dehydrogenase, cytoplasmic	P14152	36379.97	6.16	99	35.3	10/25	**299**FVEGLPINDFSR**310**	87	1393.706	3.55	1	
Mybph	Myosin binding protein H	P70402	52588.38	5.66	138	35.0	11/21	**230**SGDQDSILFIR**240**	56	1250.658	16.36	1	
Myl1	Myosin A1 catalytic light chain, skeletal muscle	P05977	20463.32	4.98	110	56.0	10/25	**81**DQGGYEDFVEGLR**93**	120	1484.7063	27.67	1	
Myl3	Myosin light chain 3	P09542	22421.56	5.03	146	55.4	12/36	**91**ALGQNPTQAEVLR**103**	126	1396.7679	9.79	1	
Mylpf	Myosin light chain, phosphorylatable, fast skeletal muscle	P97457	18824.26	4.82	226	79.9	17/26	**32**EAFTVIDQNR**41**	78	1192.596	0.19	1	

Myoz1	Myozenin 1 *	Q9JK37	31457.28	8.57	747	54.7	10	**235**MTFQMPK**241**	36	441.7267	28.20	2	
								**28**ESSGLNLGK**36**	34	452.7562	34.99	2	
								**18**LIMELTGGGR**27**	65	523.8253	61.97	2	
								**221**TAMPYGGYEK**230**	36	566.8028	88.55	2	
								**2**PLSGTPAPNKR**12**	35	379.9136	77.88	3	1
								**197**VELGIDLLAYGAK**209**	86	681.4300	62.54	2	
								**43**DVMLEELSLLTNR**55**	81	766.9457	55.58	2	
								**110**GSSGGQAGSSGSAGQYGSDR**129**	87	886.9364	69.06	2	
								**90**FLPTVGGQLETAGQGFSYGK**109**	122	1029.1402	116.26	2	
								**70**FIYENHPDVFSDSSMDHFQK**89**	32	815.0896	77.01	3	
Ndufa10	NADH dehydrogenase [ubiquinone] 1 alpha subcomplex subunit 10, mitochondrial	Q99LC3	40603.43	7.63	292	72.1	21/41	**131**LQSWLYASR**139**	50	1123.6138	21.7	1	
Ndufs1	NADH dehydrogenase (ubiquinone) Fe-S protein 1 *	Q91VD9	77182.59	5.24	675	20.5	9	**69**LSVAGNCR**76**	36	438.7373	36.14	2	
								**451**ILQDIASGR**459**	51	486.7840	13.82	2	
								**593**SATYVNTEGR**602**	48	549.2812	29.99	2	
								**646**LEEVSPNLVR**655**	50	578.3566	59.75	2	
								**88**VVAACAMPVMK**98**	49	604.8396	41.75	2	
								**201**GNDMQVGTYIEK**212**	67	677.8719	78.02	2	
								**429**VALIGSPVDLTYR**441**	70	702.4741	107.93	2	
								**185**FASEIAGVDDLGTTGR**200**	128	804.9834	107.54	2	
								**277**MHEDINEEWISDK**289**	30	549.2786	63.71	3	
Ndufs2	NADH dehydrogenase [ubiquinone] iron-sulfur protein 2, mitochondrial	Q91WD5	49229.57	5.86	176	38.7	17/33	**255**IDEVEEMLTNNR**266**	71	1462.7159	21.65	1	
Ndufs3	NADH dehydrogenase [ubiquinone] iron-sulfur protein 3, mit prec	Q9DCT2	26479.00	5.45	156	25.5	9/11	**218**VVAEPVELAQEFR**230**	99	1486.798	5.37	1	
Ogdh	Oxoglutarate dehydrogenase (lipoamide)	Q5SVX9	111839.51	6.05	247	24.2	25/34	**257**STRFEEFLQR**266**	40	1312.666	1.20	1	1
P4hb	Protein disulfide-isomerase (Prolyl 4-hydroxylase subunit beta)	P09103	55090.04	4.72	173	41.0	17/50	**84**VDATEESDLAQQYGVR**99**	130	1780.8130	12.24	1	
Pdia3	Protein disulfide-isomerase A3 *	P27773	54267.36	5.69	336	37.0	15	**83**VDCTANTNTCNK**94**	56	699.4000	0.2144	2	
								**105**IFRDGEEAGAYDGPR**119**	27	551.9000	0.0265	3	1
								**108**DGEEAGAYDGPR**119**	74	618.8000	0.0800	2	
								**120**TADGIVSHLKK**130**	37	584.9000	0.1243	2	1
								**148**FISDKDASVVGFFR**161**	50	794.4000	0.0238	2	1
								**162**DLFSDGHSEFLK**173**	45	698.3000	0.9340	2	
								**184**FAHTNVESLVK**194**	53	622.8000	0.0707	2	
								**258**DLLTAYYDVDYEKNTK**273**	29	976.0000	0.0596	2	1
								**288**TFLDAGHK**295**	27	444.8000	0.1353	2	
								**366**SEPIPETNEGPVK**378**	49	698.9000	0.0973	2	
								**415**YKELGEK**421**	32	433.8000	0.1309	2	1
								**422**LSKDPNIVIAK**432**	59	599.4000	0.0727	2	1
								**433**MDATANDVPSPYEVK**447**	52	826.9000	0.0456	2	

								**448**GFPTIYFSPANK**459**	21	671.9000	1.1090	2	1
								**482**EATNPPIIQEEKPK**495**	36	797.4000	0.0554	2	1
Pgam2	Phosphoglycerate mutase 2	O70250	28695.91	8.65	107	34.4	10/33	**11**HGESLWNQENR**21**	93	1369.639	7.04	1	
Pgk1	Phosphoglycerate kinase 1	P09411	44550.47	8.02	229	54.2	17/21	**157**LGDVYVNDAFGTAHR**171**	92	1634.8140	13.37	1	
Pgm2	Phosphoglucomutase 2	Q7TNU0	63454.38	6.02	173	31.7	17/29	**31**TQAYPDQKPGTSGLR**45**	46	1618.821	13.37	1	1
Pvalb	Parvalbumin alpha	P32848	11799.33	5.02	110	48.2	8/19	**15**AIGAFAAADSFDHKK**29**	125	1548.775	3.56	1	1
Sdha	Succinate dehydrogenase complex, subunit A	Q921P5	68032.07	6.32	228	29.7	19/26	**634**VTLEYRPVIDK**644**	40	1332.767	11.12	1	1
Serpina1c	Alpha-1-antitrypsin 1-3 *	Q00896	43335.18	5.25	267	14.3	4	**278**MQHLEQTLNK**287**	52	621.4000	130.38	2	
								**174**VINDFVEKGTQGK**186**	60	718.0000	162.96	2	
								**329**IFNNGADLSGITEENAPLK**347**	120	1002.0000	7.63	2	
								**214**KPFDPENTEEAEFHVDK**230**	35	1016.5000	31.13	2	1
Sod2	Superoxide dismutase [Mn], mitochondrial precursor	P09671	24602.93	8.80	99	42.7	8/29	**54**HHAAYVNNLNATEEK**68**	108	1710.8199	0.33	1	
Tnni2	Troponin I, fast skeletal muscle	P13412	21226.32	8.66	82	36.8	7/13	**20**SVMLQIAATELEKEESR**36**	140	1934.003	6.82	1	1
Tnnt3	Troponin T, fast skeletal muscle *	Q9QZ47	32109.66	5.26	492	19.7	5	**227**YDITTLR**233**	37	441.2518	26.76	2	
								**55**VDFDDIQK**62**	55	490.2648	50.20	2	
								**86**EEEELIALK**94**	80	537.3056	29.26	2	
								**147**ALSSMGANYSSYLAK**161**	93	781.9446	83.13	2	
								**49**IPEGEKVDFDDIQK**62**	66	816.9321	27.77	2	1
Tpi1	Triosephosphate isomerase	Q64513	26581.43	7.09	182	49.8	13/27	**7**FFVGGNWK**14**	40	954.479	4.41	1	
Tpm2	Tropomyosin 2 beta chain	P58774	32836.70	4.66	295	57.0	22/30	**168**KLVILEGELER**178**	93	1298.772	3.91	1	
Trim72	Tripartite motif-containing protein 72	Q1XH17	52816.76	6.01	157	35.2	17/37	**28**MQLQEACMR**36**	29	1166.5060	4.71	1	
Tubb4b	Tubulin beta-2C chain	P68372	49831.01	4.79	171	39.1	21/50	**36**ISEQFTAMFR**45**	74	1229.6010	2.18	1	
Tufm	Elongation factor Tu, mitochondrial precursor	Q8BFR5	49508.35	7.23	187	45.4	16/28	**91**KYEEIDNAPEER**102**	74	1492.7030	7.80	1	
Uqcrc1	Ubiquinol-cytochrome-c reductase complex core protein I, mit prec	Q9CZ13	49219.41	5.28	211	41.3	19/33	**423**RIPLAEWESR**432**	77	1256.666	6.84	1	
Vdac1	Voltage-dependent anion-selective channel protein 1	Q60932	32351.49	8.55	159	51.6	12/30	**225**YQVDPDACFSAK**236**	97	1400.625	7.07	1	
Vim	Vimentin	P20152	53556.48	5.06	330	57.4	27/36	**94**FANYIDKVR**102**	34	1125.606	0.78	1	1

2 **Supplementary Figure S1** – Full lenght immunoblot and total protein stain images of Fructose-1,6-bisphosphatase, FBP1 (A); Glutamine synthetase, GLUL (B); Glutathione synthetase, GSS (C); Beclin-1 (D); Ornithine decarboxylase, ODC (E); Microtubule-associated proteins 1A/1B light chain 3B, LC3BII(lipidated)/LC3BI(delipidated) (F); Fatty acid synthase, FASN (G); isocitrate dehydrogenase 1, IDH1 (H); and Hexokinase, HK (I).

A) FBP1 (36 kDa)

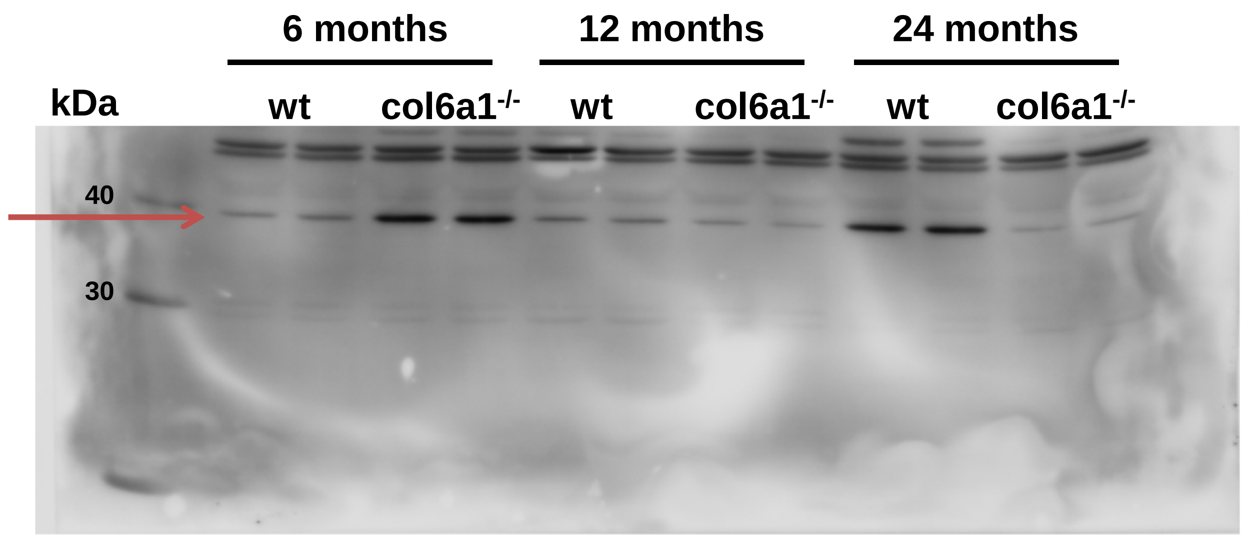

B) GLUL (43 kDa)

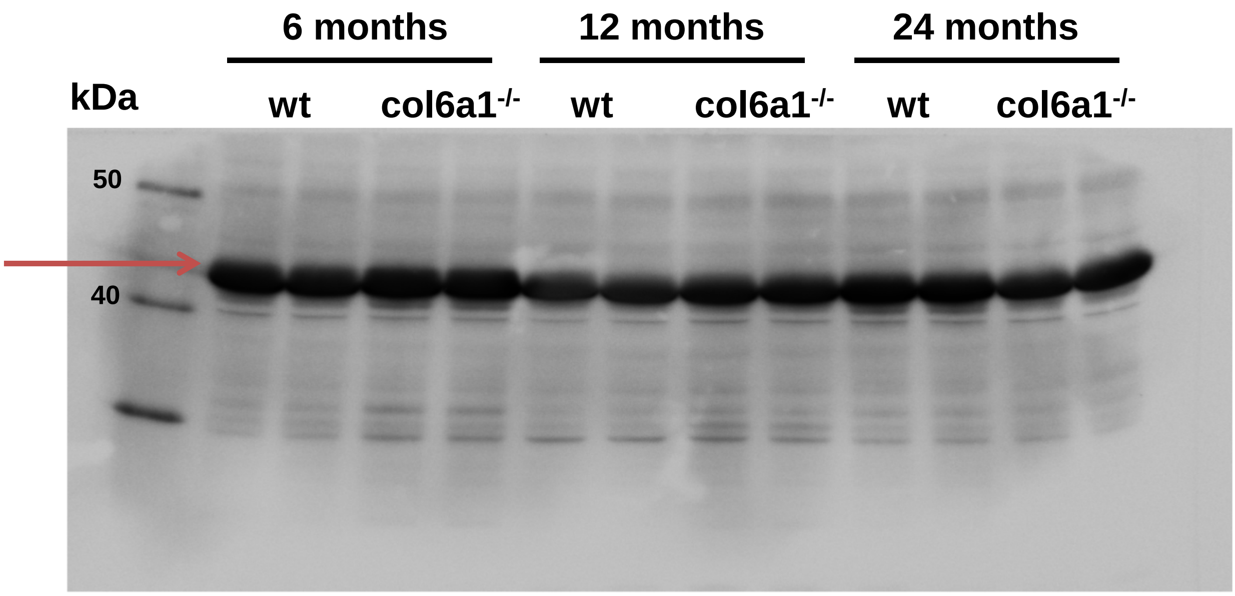

C) GSS (52 kDa)

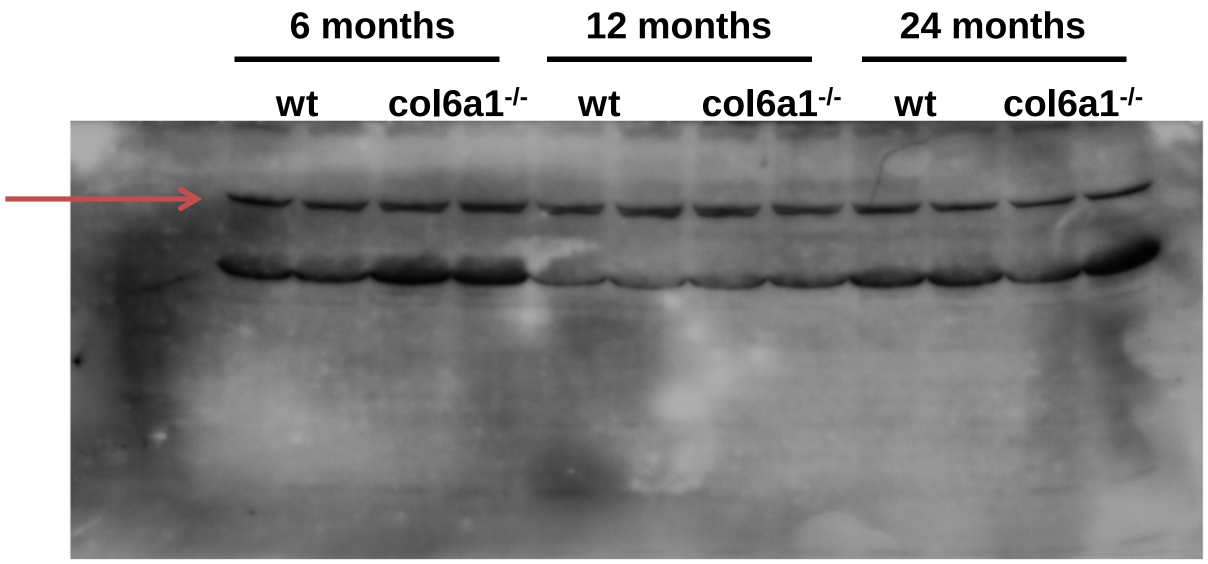

D) Beclin-1 (60 kDa)

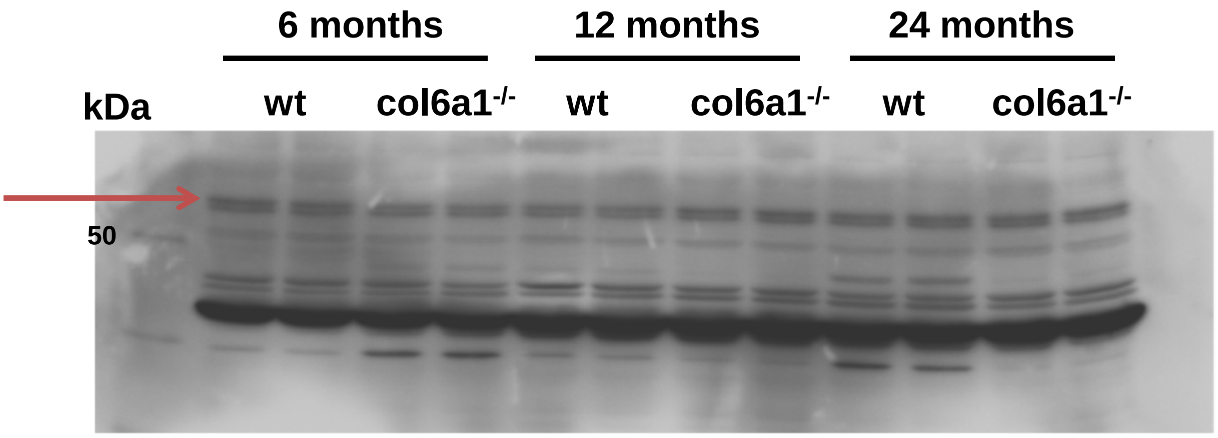

Total stain for FBP1, GLUL, GSS and Beclin1 blots

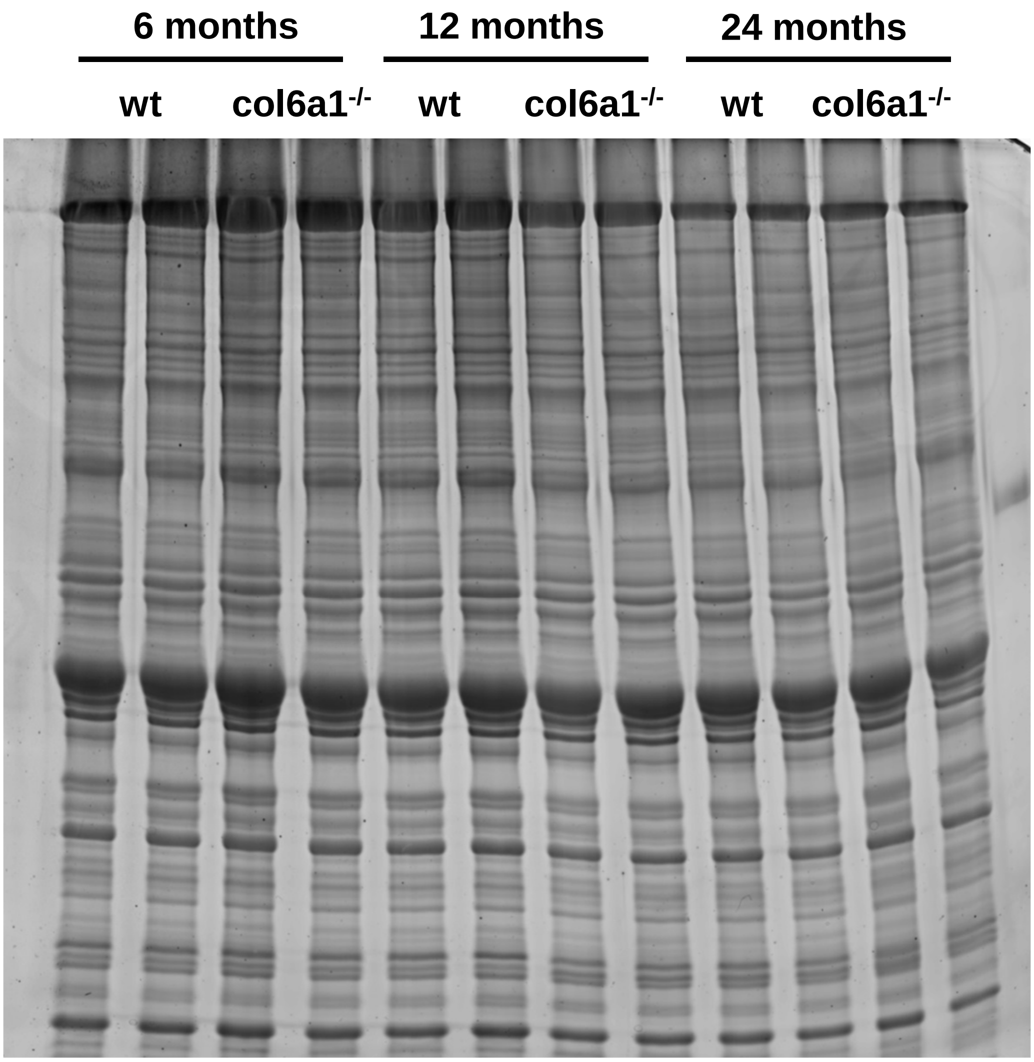

E) ODC (43 kDa)

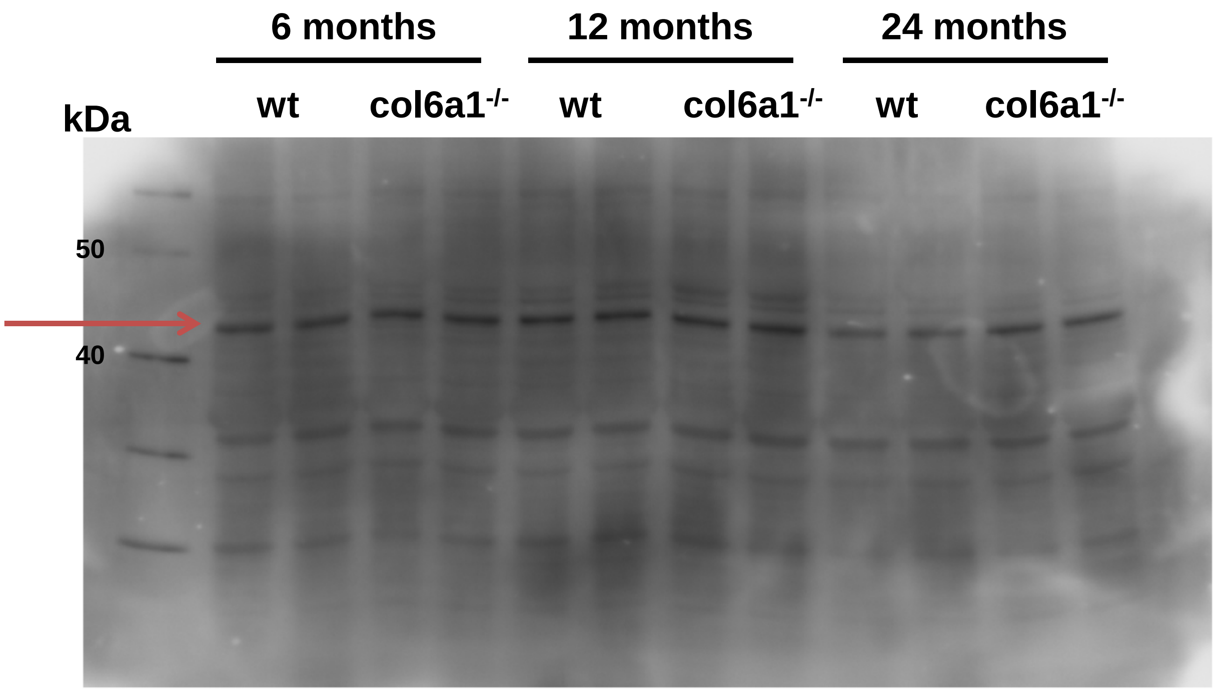

F) LC3BII/LC3BI (14-16 kDa)

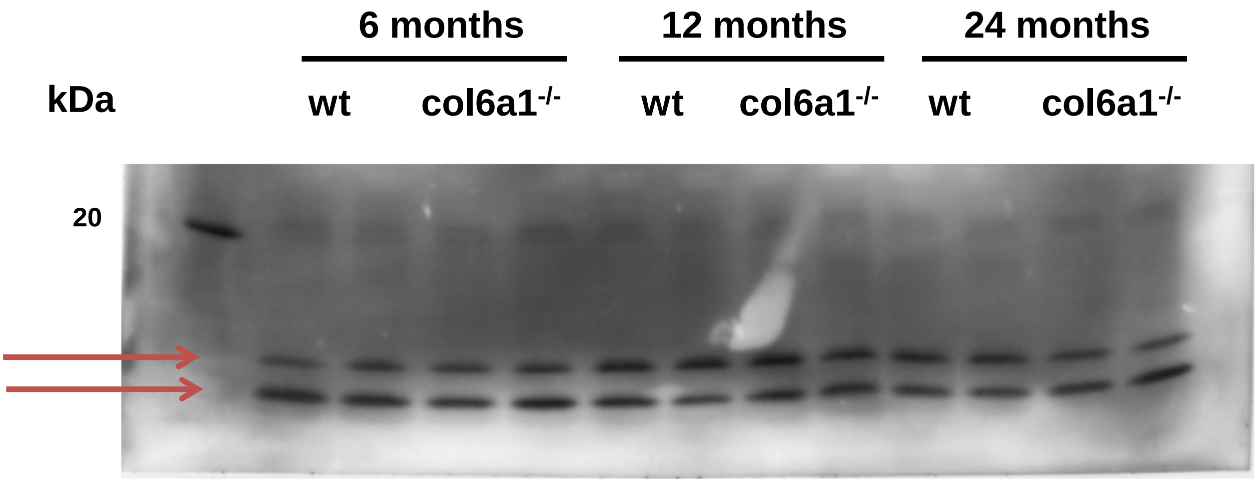

Total stain for ODC and LC3B blots

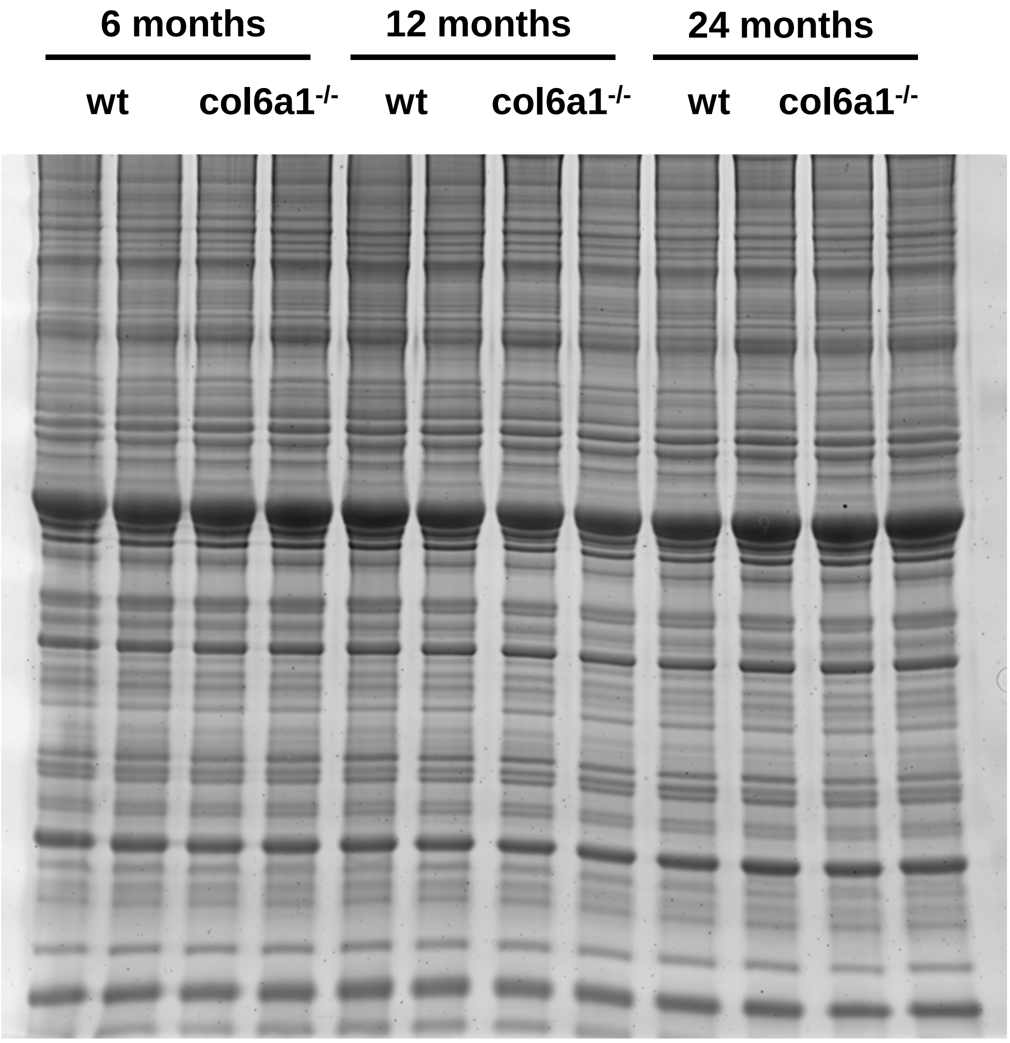

G) FASN (270 kDa)

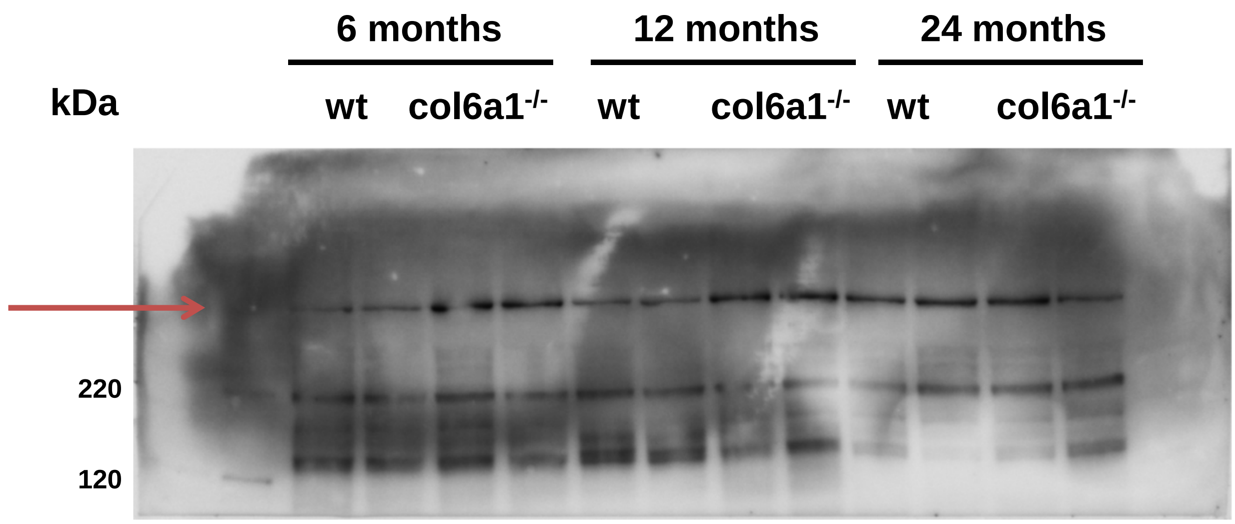

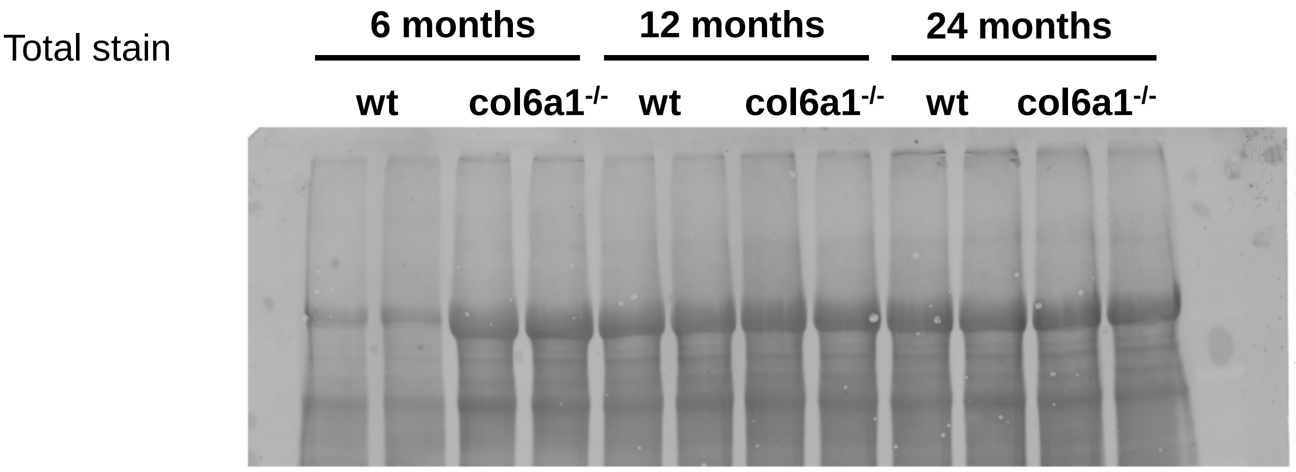

H) IDH1 (45 kDa)

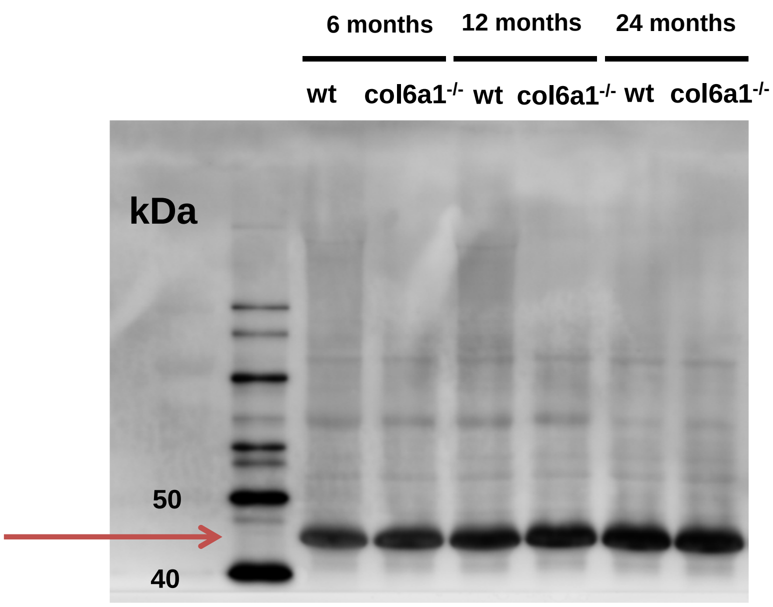

Total stain

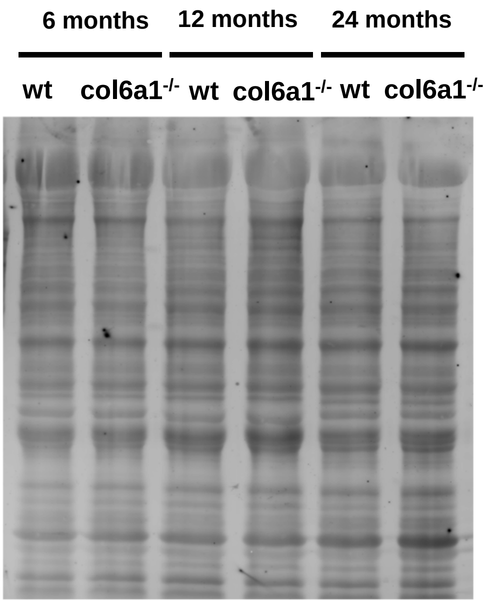

I) HK (102 kDa)

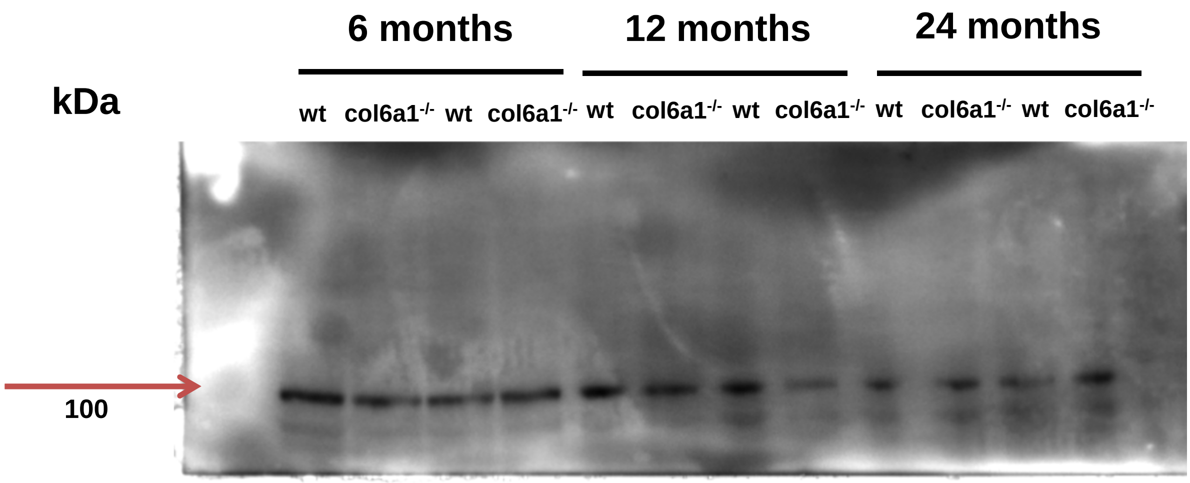

Total stain

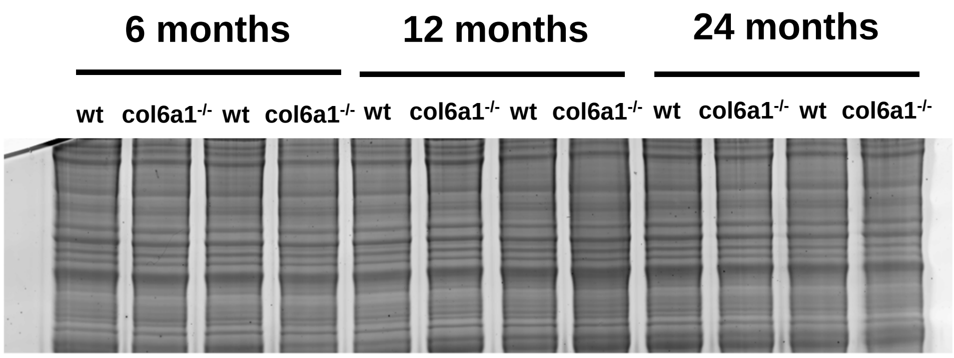

3 Supplementary Figure S2 – Oil Red O staining of muscle sections

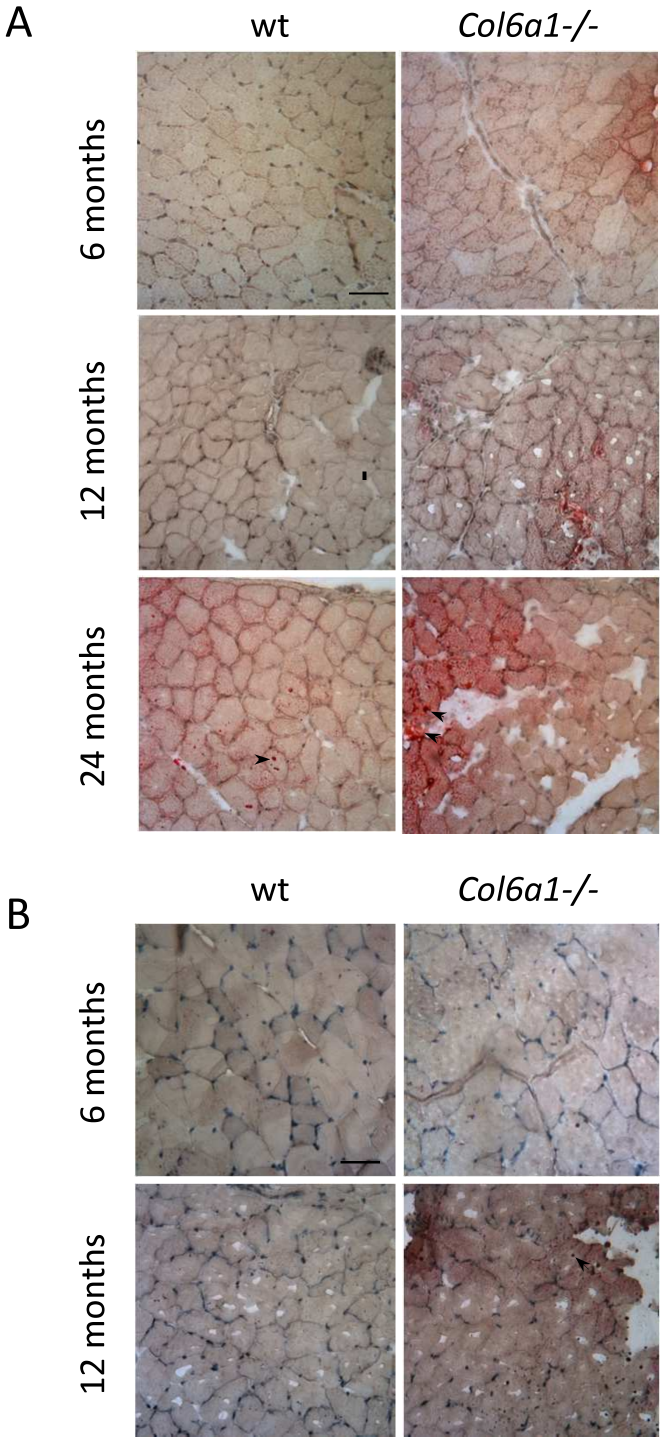

Supplementary Figure S2. Cryostat sections of diaphragm (**A**) and gastrocnemius (**B**) of wild-type and *Col6a1*$^{-/-}$ mice of 6, 12 and 24 months of age were been stained with Oil Red O staining to detect lipids deposition inside the tissue. Small red droplets appear to be evident already at 6 months in diaphragm of *Col6a1*$^{-/-}$ mice. Scale bar, 50 µm. Arrowheads indicate non-specific staining.
